# Dlk1 Is Necessary for Proper Skeletal Muscle Development and Regeneration

**DOI:** 10.1371/journal.pone.0015055

**Published:** 2010-11-29

**Authors:** Jolena N. Waddell, Peijing Zhang, Yefei Wen, Sanjay K. Gupta, Aleksey Yevtodiyenko, Jennifer V. Schmidt, Christopher A. Bidwell, Ashok Kumar, Shihuan Kuang

**Affiliations:** 1 Department of Animal Sciences, Purdue University, West Lafayette, Indiana, United States of America; 2 Department of Anatomical Sciences and Neurobiology, University of Louisville School of Medicine, Louisville, Kentucky, United States of America; 3 Division of Oncology, School of Medicine, Stanford University, Stanford, California, United States of America; 4 Department of Biological Sciences, University of Illinois at Chicago, Chicago, Illinois, United States of America; 5 Center for Cancer Research, Purdue University, West Lafayette, Indiana, United States of America; Seattle Children's Research Institute, United States of America

## Abstract

*Delta-like 1homolog* (*Dlk1*) is an imprinted gene encoding a transmembrane protein whose increased expression has been associated with muscle hypertrophy in animal models. However, the mechanisms by which Dlk1 regulates skeletal muscle plasticity remain unknown. Here we combine conditional gene knockout and over-expression analyses to investigate the role of Dlk1 in mouse muscle development, regeneration and myogenic stem cells (satellite cells). Genetic ablation of *Dlk1* in the myogenic lineage resulted in reduced body weight and skeletal muscle mass due to reductions in myofiber numbers and *myosin heavy chain IIB* gene expression. In addition, muscle-specific *Dlk1* ablation led to postnatal growth retardation and impaired muscle regeneration, associated with augmented myogenic inhibitory signaling mediated by NF-κB and inflammatory cytokines. To examine the role of Dlk1 in satellite cells, we analyzed the proliferation, self-renewal and differentiation of satellite cells cultured on their native host myofibers. We showed that ablation of *Dlk1* inhibits the expression of the myogenic regulatory transcription factor MyoD, and facilitated the self-renewal of activated satellite cells. Conversely, *Dlk1* over-expression inhibited the proliferation and enhanced differentiation of cultured myoblasts. As Dlk1 is expressed at low levels in satellite cells but its expression rapidly increases upon myogenic differentiation *in vitro* and in regenerating muscles *in vivo*, our results suggest a model in which Dlk1 expressed by nascent or regenerating myofibers non-cell autonomously promotes the differentiation of their neighbor satellite cells and therefore leads to muscle hypertrophy.

## Introduction

The paternally expressed *Delta-like 1 homolog* (*Dlk1*) gene (Entrez Gene ID 13386) lies within the imprinted *Dlk1 – Dio3* gene cluster on distal mouse chromosome 12 and encodes a transmembrane epidermal growth factor (EGF)-like protein[1]. The extracellular domain of Dlk1 can also be cleaved to generate a soluble form, called fetal antigen 1, which circulates as an abundant growth factor during development [2]. Another splice variant lacks the proteolytic cleavage site and remains constitutively membrane bound (Dlk1-C2). This membrane-bound form is most common in postnatal skeletal muscle [3], [4]. Like many imprinted genes, Dlk1 is an important regulator of mammalian development and its expression level has dramatic effects on cellular proliferation and differentiation. Elevated Dlk1 expression is associated with many tumors including acute myeloid leukemia [5], [6], [7], [8], [9], [10]. Accumulating evidence further indicates that Dlk1 is an important regulator of not only proliferation and differentiation of embryonic and adult stem cells but also functions to maintain the pluripotency of embryonic stem cells [4], [11], [12], [13], [14], [15], [16].

Recent reports indicate that *Dlk1* may play important roles in skeletal muscle development. Transcript and protein levels of Dlk1 are highest in developing fetal muscle and taper off quickly after birth [2], [17]. Interestingly, increased numbers of Dlk1**^+^** mononuclear cells and myofibers are reported in several myopathies including Becker and Duchenne muscular dystrophies which involves active muscle degeneration and regeneration [17]. Studies using transgenic mice over expressing *Dlk1* [3], [18] and callipyge mutation in sheep causing increased expression of *Dlk1* [3], [19], [20], [21] have also indicated that high levels of Dlk1 increase skeletal muscle mass in neonates. Callipyge sheep exhibit a significant increase in the size and proportion of type IIB fibers in muscles of the hind-quarters [22], [23], [24], [25]. Muscle-specific over-expression of *Dlk1* in mice yields similar results with an increase in total muscle mass and fiber diameter by six weeks of age [3]. Important roles of Dlk1 in muscle development is also evident by the observations that *Dlk1*-null mice show distinct phenotypes including increased mortality during late gestation and early neonatal development, skeletal malformations, and decreased growth rates accompanied by increased adiposity [26]. However, several key questions remain unresolved: 1) the relative role of membrane bound versus cleaved forms in muscle development and growth is unclear; 2) as Dlk1 can be cleaved and circulate, it is unknown whether muscle specific Dlk1 expression is necessary for normal muscle development and growth; 3) what role does Dlk1 play in postnatal muscle regeneration and muscle progenitor cells?

In addition, the mechanisms by which Dlk1 regulates skeletal muscle development and hypotrophy are poorly understood. Due to the EGF-like domain in Dlk1, it is classified into the same family as the Notch receptors and ligands including Delta and Serrate (Jagged) [10], [27]. Unlike Delta and Serrate, however, Dlk1 lacks the DSL (*delta/serrate/lag*) domain known to be essential for activation of Notch receptors. Therefore, Dlk1 is thought to interact with the Notch receptors through its EGF repeats as an antagonist and down-regulate signaling through Notch in *Drosophila* and Notch-1 in mammalian cell cultures [28], [29], [30]. Activation and suppression of Notch receptors by Delta occur in both *cis* (Delta suppresses Notch on the same cell; cell autonomous) or *trans* (Delta from one cell activates Notch on a neighboring cell; non-cell autonomous) manner [31]. Such distinction in signaling among progenitor cells would fit in the model of asymmetric cellular commitment seen in activated satellite cells in muscle [32]. Activated Notch signaling inhibits the formation of muscle progenitor cells in *Drosophila* [33], [34], [35] and delays the expression and activation of MyoD and myogenin, markers of muscle differentiation, in mammals [36], [37]. This suggests a model in which Dlk1 facilitates muscle differentiation through dampening of Notch signaling. However, a recent study suggests that Dlk1 does not interact with Notch1 receptor nor requires Notch activation to exert its effect on preadipocyte differentiation[38].

Satellite cells are muscle-specific stem cells that lie quiescent beneath the basal lamina of adult myofibers until needed for muscle repair. Following an injury, satellite cells proliferate and incorporate their nuclei into existing fibers while still maintaining their stem cell population. Donor satellite cells have even been shown to engraft onto recipient fibers to repair injured myofibers and ameliorate disease progression [32], [39], [40], [41], [42]. The status of a satellite cell, whether self-renewing, proliferating, or differentiating, can be determined by the expression of Pax7 and MyoD [43], [44], [45], [46], [47], [48], [49], [50], [51], [52]. We use these markers to determine the molecular mechanisms by which Dlk1 regulates fate of satellite cells during muscle development/regeneration.

To investigate the requirement of muscle specific Dlk1 in myogenic development and postnatal muscle repair, we generated a conditional *Dlk1* knock-out mouse using a *Dlk1-*floxed allele together with a *Myf5-*Cre driver. Since Myf5 is the earliest expressed myogenic regulatory factor in developing embryos and is required for myogenic determination [53], [54], this model is useful to determine how Dlk1 function in both myogenic progenitor cells and mature skeletal muscles. Our results show that muscle-specific depletion of Dlk1 leads to defective muscle development, elevated inflammatory responses, and delayed muscle regeneration. We further provide evidence that Dlk1 non-cell autonomously affects satellite cell fate choice between self-renewal and differentiation.

## Results

### Defective muscle formation, growth retardation, and altered myosin heavy chain gene expression in muscle-specific Dlk1 mutant mice

The role of membrane-bound Dlk1 in skeletal muscle was investigated by creating a conditional mutant mouse model. We used the *Myf5-Cre* mouse to inactivate Dlk1 in the myogenic lineage [53], [54] of *Dlk1^flox^* mice. As Dlk1 is an imprinted gene expressed only from paternal allele [1], paternal heterozygous *Myf5-*Cre/*Dlk1* ^+(m)/flox(p)^ displayed identical Dlk1 expression profiles and phenotypes as the homozygous *Myf5-*Cre/*Dlk1* ^flox/flox^ alleles (data not shown). Thus, *Dlk1^flox/+^* males were crossed with *Myf5-Cre* females to produce paternal heterozygotes for muscle-specific *Dlk1* knockout (henceforth *Dlk1* cKO).

**Figure 1:**
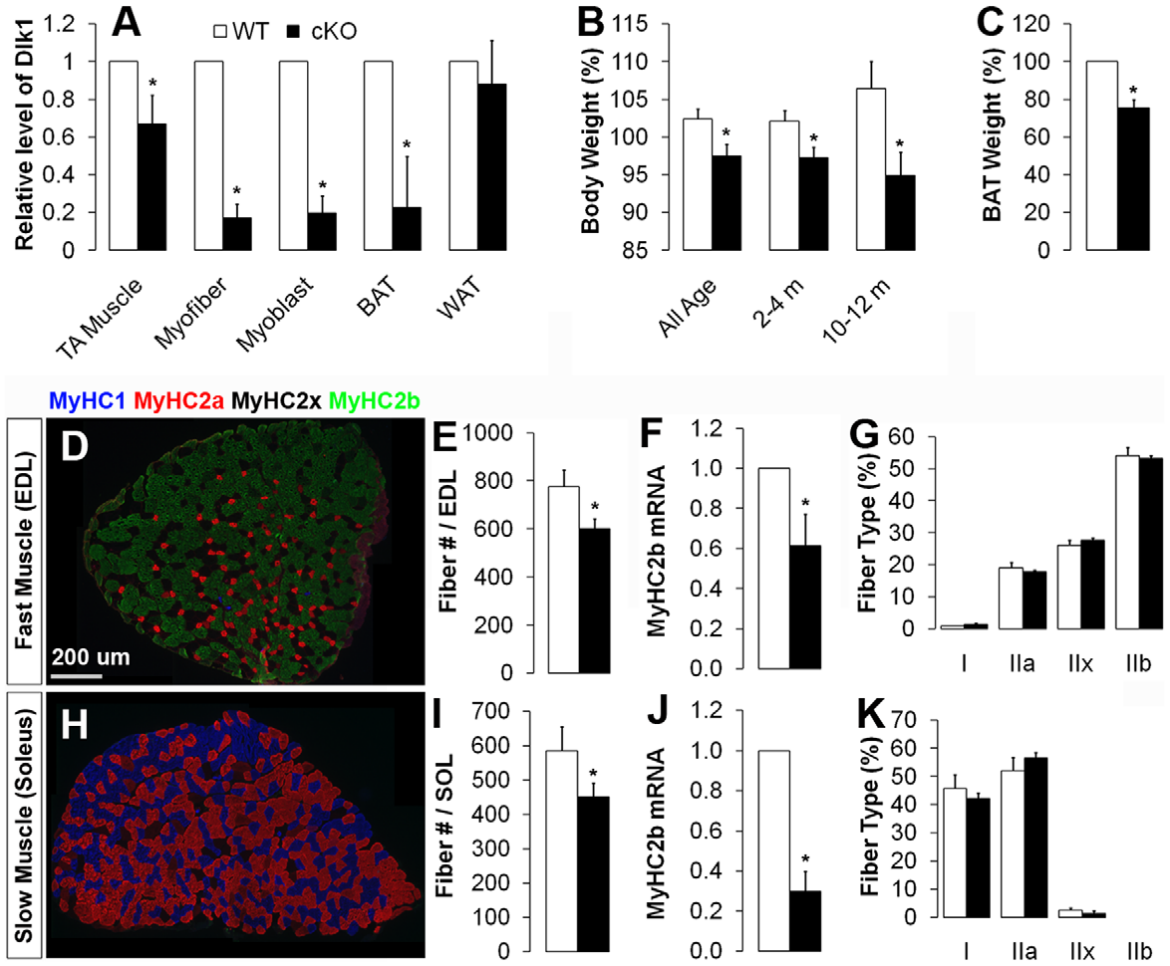
Myf5-Cre mediated mutation of paternal *Dlk1*(cKO) results in defects in muscle formation and growth. Asterisks in all graphs denote p<0.05compared to wild-type (WT) controls. **A**: Quantitative PCR confirming muscle-specific *Dlk1* knockout in cKO mice in whole muscle, myofibers, myoblasts and BAT. **B**: Relative body weight (BW) of WT (n = 17, 9, 4 for all age, 2–4 month and 10–12 month mice, respectively) and cKO (n = 18, 9, 5) littermates. **C**: Relative mass of BAT of WT (n = 3) and cKO (n = 3) mice. **D–K**: Muscle fiber composition in representative fast-twitch (EDL) and slow-twitch soleus (SOL) muscles revealed by MyHC isoform-specific antibodies and qPCR. **D**: MyHC isoform staining of representative EDL. Total myofiber number for EDL(**E**) and SOL (**I**) muscle in WT and cKO (n = 4 pairs). *Myosin heavy chain (MyHC) IIB* gene expression in EDL (**F**) and SOL (**J**;n = 3 pairs) muscles. Percent of each MyHC isoform by immunostaining in EDL (**G**) and SOL (**K**; n = 3 pairs).

By performing qPCR assays we found ∼35% reduction in *Dlk1* expression in whole muscles of Dlk1 cKO, suggesting that Dlk1 expressed by myofibers only accounts for about one-third of the total *Dlk1* mRNA expressed by the whole muscle (Fig. 1A). These results are in agreement with our immunohistological results that Dlk1 is mainly expressed by interstitial non-myogenic cells within the muscle (Supplementary Fig. S1). To confirm muscle-specific knockout of *Dlk1,* we isolated single myofibers from EDL muscles and quantified *Dlk1* expression, which was reduced by ∼90% in the *Dlk1* cKO myofibers (Fig. 1A). The residual (10%) *Dlk1* expression detected in isolated *Dlk1* cKO myofibers probably originated from contaminating interstitial cells attached on the fibers[44]. Furthermore, *Dlk1* levels in *Dlk1* cKO brown adipose tissue (BAT) were reduced by 80%, again in agreement with our recent report that the brown adipose tissue is derived from a *Myf5-*lineage[55]. In contrast, white adipose tissue, known to be derived from Myf5-independent lineages[55], exhibited no changes in *Dlk1* expression. These results confirm specific deletion of *Dlk1* in muscle and brown fat lineage mediated by the Myf5-Cre allele.

The *Dlk1* cKO mice were born at expected Mendelian ratios (Expected ratio: 25%; observed ratio 25.9±3.9%, n = 17 litters) and appeared to be healthy and behaviorally normal. However, notable phenotypic differences were observed in the *Dlk1* cKO mice compared to wild-type siblings. First, the mutants displayed a significant reduction in body weight (Fig. 1B); this reduction appeared to be more prominent at older age (11% reduction in old vs 5% in young mice, however p = 0.34). The brown fat mass also decreased by 20% in the *Dlk1* cKO mice (Fig. 1C). To further determine if the observed body weight loss was mainly due to reduced muscle mass, we compared the number of muscle fibers in representative fast (EDL, Fig. 1D) and slow (soleus, Fig. 1H) muscles. Indeed, there is a roughly 25% reduction in total myofiber numbers in the cKO in both muscle types (Fig. 1E & I), which likely contributes to the 5–10% decrease in body mass, as skeletal muscles generally account for about 40% of total body weight. As myofiber numbers are known to be fixed at birth[56], the reduced number of myofibers in cKO reflects defects in embryonic myogenic development. Together, these observations suggest that our *Dlk1*conditional mutation leads to defective muscle development and postnatal growth retardation.

Over-expression of *DLK1* in Callipyge sheep muscle is linked to a switch in fiber type towards fast twitch glycolytic fibers that express the *myosin heavy chain (MyHC) IIB* gene. We therefore examined if the deletion of *Dlk1* leads to fiber type switching. A marked reduction in mRNA levels of *MyHC IIB* was detected in the *Dlk1* cKO mice in both EDL and SOL muscles (Fig. 1F & J), whereas the expression of other *MyHC* genes were not affected (not shown). MyHC isoform-specific monoclonal antibody labeling of EDL and SOL muscles, however, did not indicate any differences in the proportion of each fiber type (Fig. 1G & K). Therefore, muscle-specific ablation of *Dlk1* appears to reduce the levels of fast type IIB MyHC, but does not lead to fiber type switching in mice.

### Delayed muscle regeneration in *Dlk1* cKO

**Figure 2:**
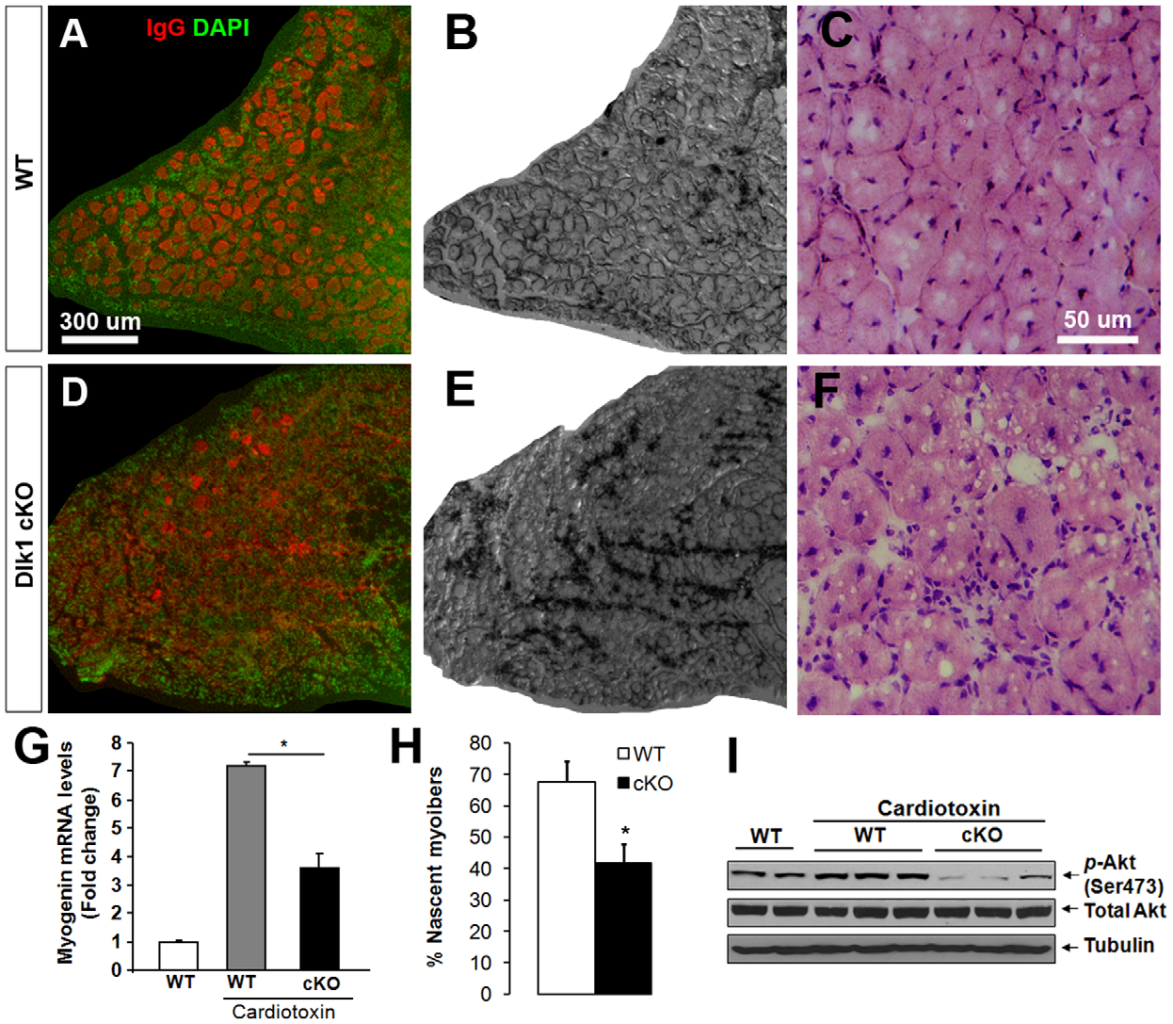
Muscle-specific *Dlk1* cKO results in impaired muscle regeneration after injury. **A–F**: Morphology of TA muscle 1 week after cardiotoxin injury in WT (**A–C**) and cKO mice (**D–F**). **A**, **D**: Fluorescent immunostaining of mouse IgG showing injured fibers in red and Dapi nuclei staining in green. **B**, **E**: Brightfield images showing increased fibrosis and scarification (Dark signal due to poor light penetration) in cKO mutant muscles. **C**, **F**: H–E staining showing poor organization and increased infiltration of non-myogenic cells in mutant muscles. **G**: Myogenin mRNA levels increase after injury, but cKO mice have an impaired myogenin response (n = 4). **H**: Total number of nascent fibers (n = 7 pairs) are decreased in cKO injured muscles. **I**: Levels of phosphorylated Akt are decreased in cKO muscle compared to WT control and injured muscles.

To investigate how Dlk1 deficiency may affect adult myogenesis, The tibialis anterior (TA) muscles of *Dlk1* cKO and wild-type mice were given intramuscular injections of CTX and the regenerated muscles were examined at different time points. Regeneration of the TA muscle was considerably impaired in the *Dlk1* cKO at 5–7 days after injury, a time point at which regeneration peaked in the wild-type mice. Skeletal muscle regeneration involves the repair of existing degenerated fibers and *de novo* formation of new fibers. Existing fibers undergoing active repair can be readily identified by their large diameter with centrally localized nuclei and non-specific IgG binding due to immune cell infiltration (Fig 2A). *De novo* formed new fibers are considerably smaller in diameter with central nuclei. While wild-type muscles regenerated uniformly with little fibrosis and scarification (Fig. 2A–C), the *Dlk1* cKO muscles were poorly regenerated with extensive fibrosis, scarification, and interstitial space occupied by massive cellular infiltration (Fig. 2D–F).

The defective regeneration of *Dlk1* cKO fibers is substantiated by a significant decrease in myogenin mRNA expression levels (Fig. 2G) and a roughly 25% decrease in nascent *de novo* fiber formation (Fig. 2H) five days after injury. In addition, phosphorylated Akt (the activated form) levels were decreased in the cKO (Fig. 2I), suggesting an impairment of the Akt/mTOR signaling pathway that is known to promote protein synthesis and myotube hypertrophy [57]. This observation is consistent with our previous finding that Akt signaling is enhanced in *DLK1* over-expressing callipyge sheep [58]. Together, these data suggest that Dlk1 is necessary for proper skeletal muscle regeneration in adult mice.

### Elevated inflammatory response to injury in *Dlk1* cKO muscles

**Figure 3:**
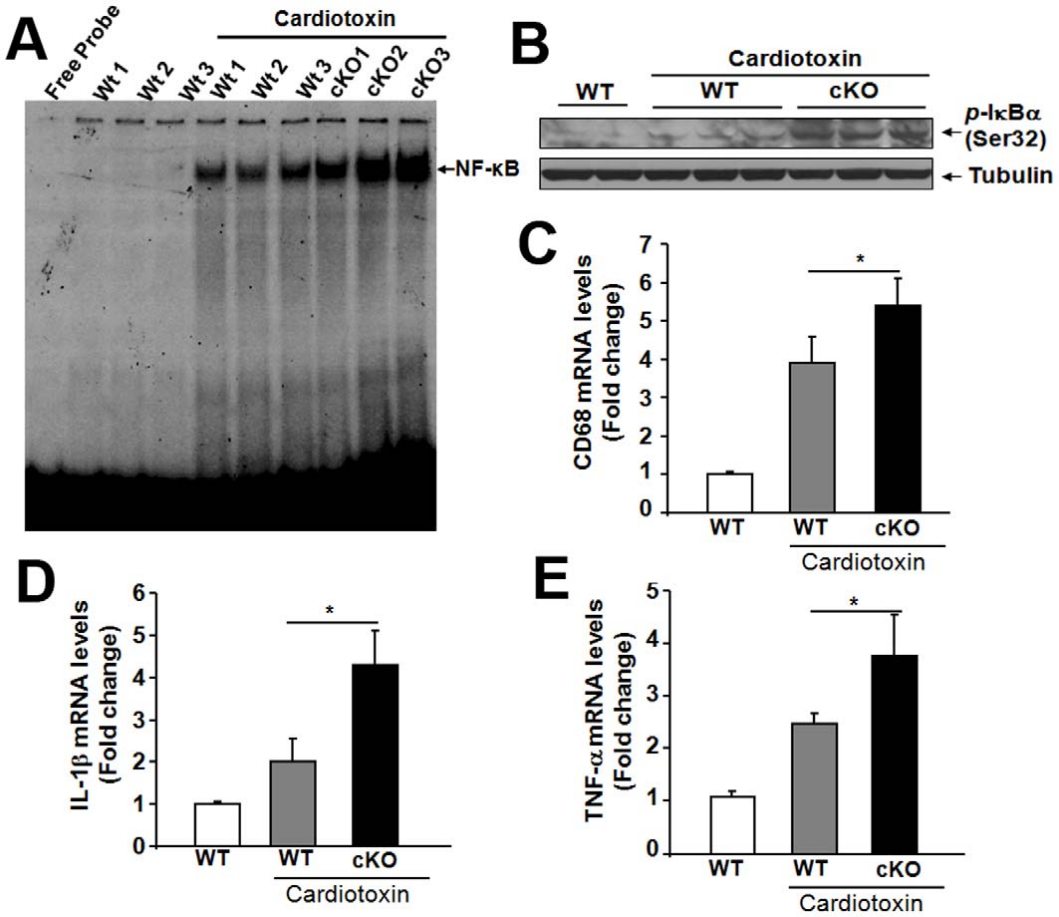
Muscle-specific *Dlk1* cKO results in increased inflammation and NF-κB signaling after injury. **A–B**: NF-κB pathway is up-regulated in cKO injured muscle as indicated by increase DNA binding by nuclear NF-κB (**A**) and phosphorylated Iκ-Bα (**B**). **C**: CD68 (macrophage marker) mRNA levels are increased in cKO injured muscles (n = 4). **D–E**: Pro-inflammatory cytokines IL-1β (**D**) and TNF-α (**E**) are up-regulated in cKO injured muscles (n = 4).

As muscle injury causes an inflammatory immune response within the damaged tissue [59],we examined whether aberrant inflammatory responses contributed to the defective regeneration of the *Dlk1* cKO muscles. The increased interstitial nuclei density in regenerating cKO muscles (Fig. 2F) indicates excessive infiltrated macrophages, which was confirmed by the high mRNA levels of CD68 (Fig. 3C), a cell surface marker for macrophages. Nuclear factor kappa B (NF-κB) is a major pro-inflammatory transcription factor controlling the expression of a plethora of genes involved in inflammation and immune responses [60]. Several published reports have suggested that NF-κB inhibits myogenesis at multiple levels including suppression of MyoD [61], [62]. Moreover, it has been found that muscle-specific inhibition of NF-κB dramatically improves skeletal muscle regeneration in response to CTX-mediated injury [63]. We therefore sought to investigate whether depletion of *Dlk1* affects the activation of NF-κB transcription factor in regenerating muscles. DNA-binding activity of NF-κB in regenerating muscle was found to be markedly higher in *Dlk1* cKO mice compared to wild-type mice (Fig. 3A). The increased activation of NF-κB pathway was also evident by our results that the levels of phosphorylated IκBα was up-regulated in regenerating muscle of *Dlk1*cKO mice compared to those of wild-type mice (Fig. 3B). Furthermore, the expression of NF-κB-regulated pro-inflammatory cytokines IL-1β and TNF-α was also significantly higher in myofibers of *Dlk1*cKO mice compared to wild-type mice in response to injury (Fig. 3D–E). Both TNF-α and IL-1β have been previously shown to inhibit myogenesis [62], [63], [64]. Collectively, these data suggest that the loss of *Dlk1* exacerbates the inflammatory response and augments the expression of inflammatory cytokines which may be responsible for the reduced myofiber regeneration after injury.

### *Dlk1* ablation leads to reduced MyoD expression and facilitates self-renewal of activated satellite cells

**Figure 4:**
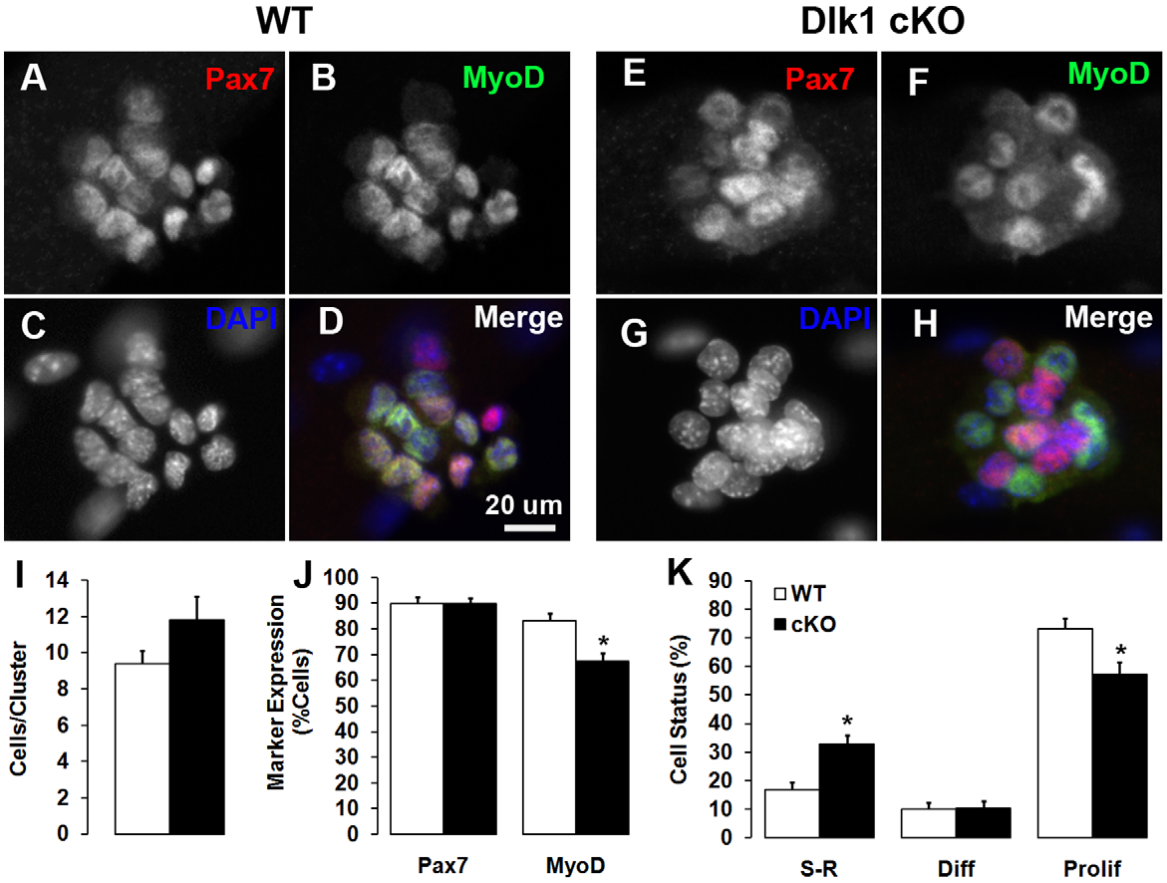
Dlk1 regulates satellite cell self-renewal and differentiation. **A–D**: Pax7 and MyoD expression in a representative cluster of myoblasts on a wild-type EDL fiber after 3 days of culture. Pax7^+^/MyoD^−^, Pax7^+^/MyoD^+^ and Pax7^−^/MyoD^+^ cells represent self-renewing, proliferating and differentiating cells, respectively. **E–H**: A representative cluster of myoblasts on a cKO fiber cultured under identical culture conditions. **I**: The average number of cells per cluster. **J**: The percentage of cells expressing Pax7 and MyoD (note that most cells co-express Pax7 and MyoD; see **K** below). **K**: The proportion of cells at three different statuses: self-renewal (**S–R**; Pax7^+^/MyoD^−^), differentiation (**Diff**; Pax7^−^/MyoD^+^) and proliferation (**Prolif**; Pax7^+^/MyoD^+^). In **I–K**, n = 34 for WT, and n = 27 for cKO; * indicates p<0.05 compared to WT.

The defective muscle regeneration suggests that our muscle-specific *Dlk1* mutation may also affect the normal function of satellite cells, which underlies muscle regeneration. To investigate this possibility, the potential effects of Dlk1 on satellite cell self-renewal and differentiation were assessed *ex vivo* by activating quiescent satellite cells attached to single EDL myofibers in culture. In this model, satellite cells initially only express Pax7, then activate MyoD, and enter the cell cycle. Proliferating cells then either down-regulate Pax7 in order to differentiate, or down-regulate MyoD and self-renew[43], [45], [51]. After 3 days in culture, distinctive clusters of myoblasts are readily detectable on myofibers. Myoblast clusters were stained for Pax7 and MyoD so that Pax7^+^/MyoD^−^, Pax7^+^/MyoD^+^, and Pax7^−^/MyoD^+^ cells represent self-renewing, proliferating, and differentiating cells, respectively (Fig. 4A–H).

The average number of cells per cluster was not significantly different between wild-type and conditional mutants (Fig. 4I). In addition, the percentage of cells expressing Pax7 was not affected in the cKO compared with the wildtype cells (Fig. 4J). However, a significant reduction of cells expressing MyoD was observed in the cKO cells (Fig. 4J), leading to a shift in the cell fate status. Specifically, the proportion of self-renewing cells (Pax7^+^/MyoD^−^) was increased in the Dlk1 cKO while proliferating cells (Pax7^+^/MyoD^+^) were decreased (Fig. 4K). These results suggest that the lack of Dlk1-initiated signaling promotes satellite cell fate choice towards the self-renewal state as the expense of proliferation.

### Over-expression of Dlk1 promotes myoblast cell cycle withdrawal and differentiation

**Figure 5:**
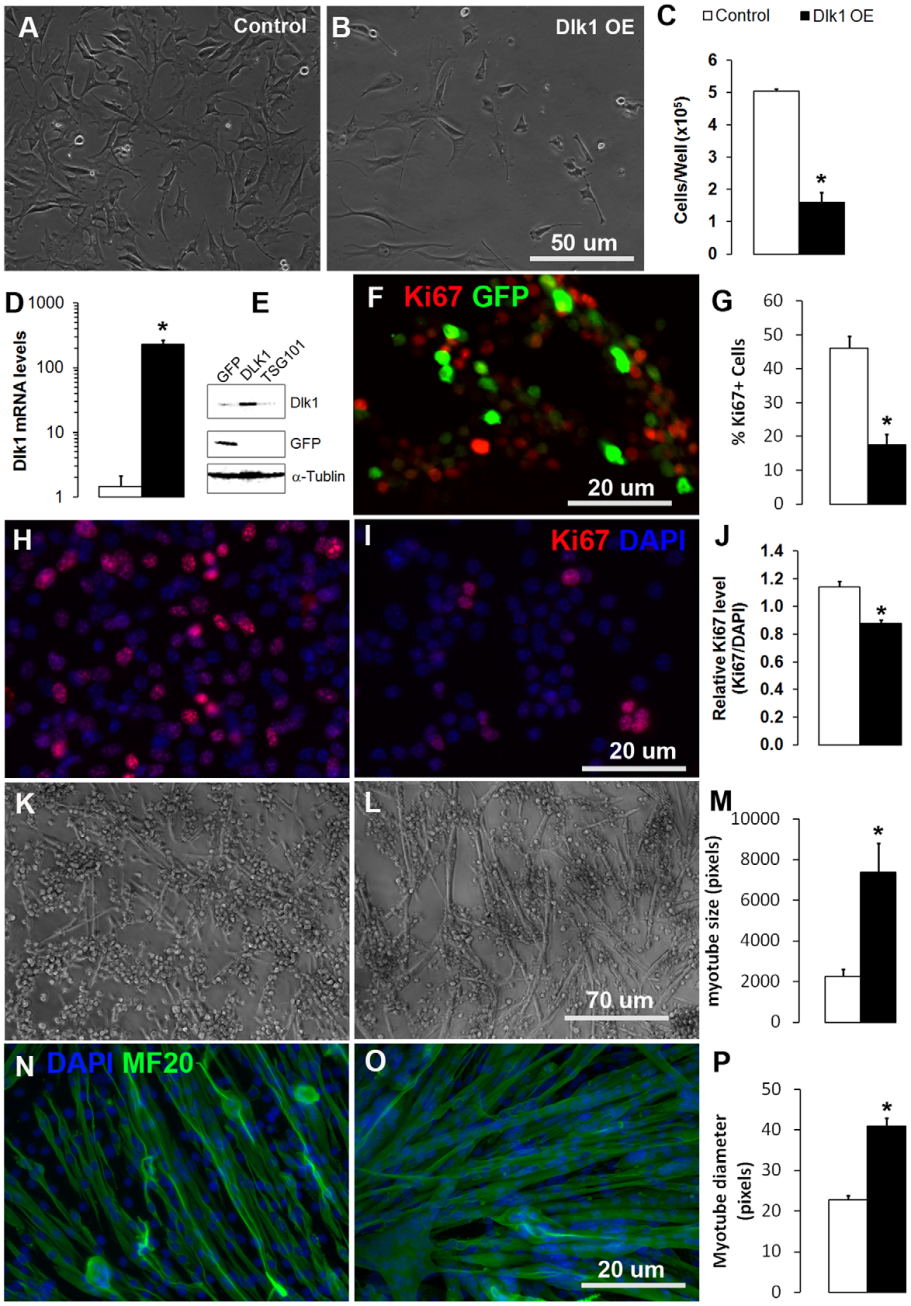
*Dlk1* over-expression (OE) or recombinant Dlk1 protein inhibits proliferation but promotes differentiation of myoblasts. **A–B**: *Dlk1* over-expression in C2C12 myoblasts. Cells were transfected with either GFP (**Control**; **A**) or **Dlk1 OE** (**B**) plasmids and cultured for 3 days. C: Cell numbers of **Control** and **Dlk1 OE** at day 3 after transfection. **D–E**: Relative *Dlk1* mRNA levels in the **Control** and **Dlk1 OE** cells measured by quantitative Realtime PCR (n = 4). **E**: Protein levels of Dlk1, GFP and α-tubulin in GFP control transfected cells (**GFP**), Dlk1 over-expression cells (**DLK1**), and cells transfected with an unrelated negative control gene (**TSG101**). **F**: Primary myoblasts co-transfected with Dlk1 and GFP (4∶1) plasmids were cultured for 2 days and labeled with a cell proliferation marker Ki67 together with GFP staining. GFP signals (indicating Dlk1 positively transfected cells) exhibit little colocalized with Ki67 immunofluorescence. **G**: Percentage of cells displaying Ki67 staining in **Control** and **Dlk1 OE** cells (n = 3). **H–J**: Primary myoblasts transfected with an empty plasmid (H, control) or Dlk1 plasmid (**I**, **OE**) cultured for 3 days in growth medium and labeled with Ki67 (in red) and DAPI (in blue). The average Ki67 immunofluorescenc intensity was measured with Photoshop and normalized to DAPI intensity (**J**, n = 3 per treatment). **K–M**: Phase-contrast images of primary myoblasts differentiated for 48 hrs after transfected with either empty plasmids (**K**) or Dlk1 OE plasmids (**L**). **M**: Average pixel size of myotubes in **Control** and **Dlk1 OE** treatments as shown in **H&I** (n = 20 random tubes measured). **N–O**: Primary myoblasts grown on Matrigel plus vehicle control (**N**) and on Matrigel plus Dlk1 recombinant protein (**O**) after 3 days in differentiation medium. **Green** fluorescence (**MF20**) marks sarcomere myosin heavy chain and **Blue** is **DAPI** counterstaining for nuclei. **P**: The relative diameters of the resulting myotubes as shown in **N & O** were measured with Image J software. Control is the open bar (n = 66 myotubes) and Dlk1 recombinant protein treated cells are represented by the solid black bar (n = 66 myotubes). Asterisks in all bar graphs indicate p<0.05 compared to control groups by student t-test.

To directly test how Dlk1 regulates myoblasts, we over-expressed the membrane-bound form of *Dlk1* in C2C12 and primary myoblasts using the Neon transfection system (Invitrogen, Inc.), which gives 50–75% transfection efficiency in our hands. We first over-expressed Dlk1 in C2C12 myoblasts (Fig. 5A–B). Strikingly, C2C12 cells over-expressing *Dlk1* failed to expand and the cell number at day 3 after transfection was only 30% that of the GFP (N1-GFP, Clontech) transfected control (Fig. 5C). To confirm that Dlk1 is indeed over-expressed, we measured the mRNA and protein levels of Dlk1 in control and *Dlk1* transfected cells. *Dlk1*-transfected C2C12 cells expressed 230 times more mRNA and much higher protein levels of Dlk1 compared to control cells transfected with GFP (Fig. 5D–E). Similar reductions in cell numbers were observed in satellite cell-derived primary myoblasts over-expressing *Dlk1* (Data not shown). To investigate if the observed reduction in cell number is due to an inhibition of cell proliferation, we co-transfected primary myoblasts with 4∶1 ratio of *Dlk1* and *GFP* plasmids and measured cell proliferation with Ki67, a well-established cell proliferation marker (Fig. 5F). Notably, Ki67 expression was found to be rarely co-localized to the GFP^+^ cells (Fig. 5F), which should also be positively transfected with *Dlk1* due to the much higher (4x) concentrations of *Dlk1* plasmids used during transfection. Quantification showed that the percentages of Ki67^+^ cells in GFP^+^ cells was only 17.5%, compared to 46.2% in GFP^−^ cells (Fig. 5G). To further confirm this observation, we examined Ki67 expression in primary myoblasts electroporated with empty plasmid (Fig. 5H) or Dlk1 plasmid (Fig. 5I). We found that the average intensity of Ki67 immunofluorescene (normalized to DAPI fluorescence) is significantly reduced in the Dlk1 over-expressing cells (Fig. 5J). These results demonstrate that Dlk1 over-expression inhibits myoblast proliferation.

Strikingly, primary myoblasts over-expressing *Dlk1* displayed accelerated differentiation kinetics. At confluent cell density, it typically takes 4 days before primary myoblasts are fully differentiated. At 2 days after induced differentiation by serum withdrawal, only few myotubes were detectable in the control cells (Fig. 5K), whereas Dlk1 over-expressing cells readily differentiate and numerous myotubes were detectable at the same time point (Fig. 5L). Additionally, the average size of the myotubes formed was significantly larger in the Dlk1 over-expressing cells (Fig. 5M), and the expression of sarcomere myosin heavy chain, revealed by MF20 immunofluorescence, is roughly 2.5 times higher in the *Dlk1* over-expressing cells (Data not shown). These observations provide direct evidence that Dlk1 promotes myogenic differentiation.

As extracellular ligands such as Dlk1 commonly exerts their effects on neighbor cells in a cell-contact dependent manner, we hypothesized that Dlk1 over-expression non-cell autonomously regulates myoblast proliferation and differentiation. To confirm that the observed effect of Dlk1 on myoblast differentiation is non-cell autonomous, we examined myoblast growth and differentiation in the presence of Dlk1 coated substrate. Wild-type myoblasts were allowed to proliferate and differentiate on a bed of Matrigel (BD Biosciences) containing vehicle control (Fig. 5N) or 500 ng/ml of recombinant Dlk1 protein (Fig. 5O). Cells differentiated in the presence of recombinant Dlk1 had reduced Ki67 expression (Data not shown) but formed significantly larger myotubes than cells grown under control conditions (Fig. 5P). These data, combined with the loss-of-function analysis, provide strong evidence that high levels of exogenous Dlk1 promote cell cycle withdrawal and differentiation of myoblasts into myotubes.

### Dlk1 upregulation correlates to myoblast differentiation and muscle regeneration *in vivo*

**Figure 6:**
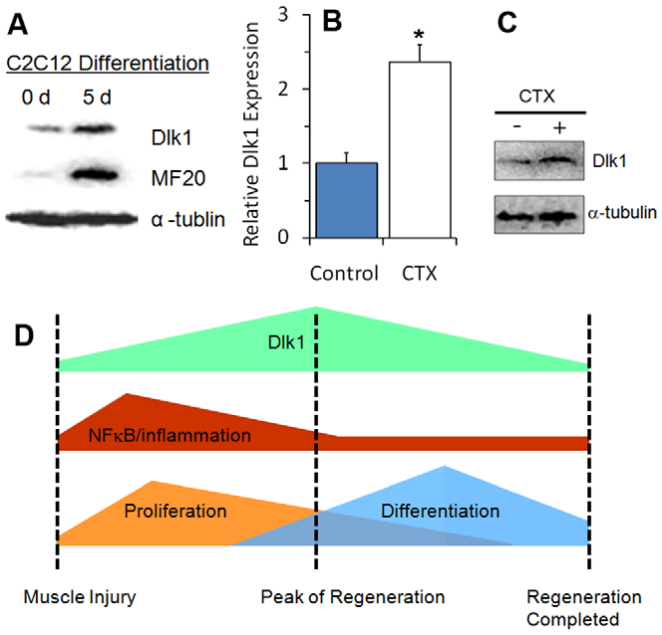
Dlk1 up-regulation correlates to myogenic differentiation and skeletal muscle regeneration after injury. **A**: Representative Western-blot image showing that Dlk1 protein levels increase 5 days after serum-withdrawal induced differentiation of C2C12 myoblasts. **B**: The relative level of *Dlk1* mRNA in the TA muscle revealed by quantitative Realtime PCR 5 days after cardiotoxin-induced regeneration (* indicates P<0.05; n = 18 for control TA; 6 for CTX treated TA). **C**: Dlk1 protein expression revealed by Western-blot in resting and regenerating TA muscles 5 days after cardiotoxin treatment. **D**: A model depicting how Dlk1 level varies during muscle quiescence and regeneration, which correlates to suppression of NK-κB activity and the onset of myoblast differentiation.

Our data establish a non-cell autonomous role of Dlk1 in myogenic differentiation. To determine the physiological relevance of this observation, we examined whether Dlk1 upregulation correlates to myoblast differentiation. Indeed, Dlk1 protein expression, along with myosin heavy chain protein expression, is upregulated in newly differentiated myotubes 5 days after induction of differentiation (Fig. 6A). In addition, both mRNA (Fig. 6B) and protein (Fig. 6C) levels of Dlk1 were significantly upregulated 5 days after CTX-induced muscle regeneration in vivo. In contrast, Dlk1 protein levels were very low in both quiescent and activated satellite cells, but elevated in newly regenerated myofibers (Supplementary Fig. S1A&C). Similarly, cultured myoblasts expressed much lower levels of *Dlk1* mRNA in relative to isolated myofibers or whole muscles (Supplementary Fig. S1D). Combined with our earlier results from *Dlk1* cKO, these results indicate that Dlk1 upregulation is correlated and necessary for normal differentiation of muscle progenitor cells.

Based on our findings and previous observations, we propose a model in which the timing of Dlk1 critically affect muscle regeneration after injury (Fig. 6D). Upon muscle injury, satellite cells are activated and proliferate. Dlk1 level in the whole muscle peaks at the height of satellite cell proliferation (Fig. 6A–C), allowing many of the proliferating myoblasts to differentiate and repair the muscle damage. As Dlk1 levels later drop[17], a portion of undifferentiated cells then return to the self-renewed state, or quiescence. The level of Dlk1 available on neighboring tissues during satellite cell activation determines, in a non-cell autonomous manner, the fate of the satellite cells in question. High levels of Dlk1 promote higher rates of differentiation while low levels trigger more self-renewing cells. However, our model does not exclude a potential cell-autonomous role of Dlk1 in myoblast differentiation. Finally, our *Dlk1*cKO mice had significantly higher levels of NF-κB activation, pro-inflammatory cytokines, and macrophage infiltration. Thus endogenous Dlk1 may also be required to limit the inflammatory response in regenerating muscles.

## Discussion

Delta-like 1 (Dlk1) exists in both a cleaved circulating form and a constitutively membrane bound form and is crucial for the proper development of several tissues in mammals. However, the specific functions and signaling mechanisms of the predominantly membrane-bound form (Dlk1-C2) in skeletal muscle and muscle progenitor cells remain unclear. Previous reports have indicated that muscle-specific over-expression of *Dlk1* causes postnatal muscle hypertrophy in both mice and sheep [20], [21], [65], [66]. However, the phenotype in sheep is complicated as the callipyge mutation also alters the expression of several other imprinted genes near the *Dlk1* allele [19], [20], [21], [66], [67], [68], [69], [70]. In addition, constitutive Dlk1 knockouts and over-expression are mostly embryonic or perinatal lethal[18], [26], [71]. This study was thus designed to elucidate the role of local non-circulating Dlk1 in skeletal muscle development and regeneration by creating a muscle-specific *Dlk1* knock-out mouse (*Dlk1* cKO). Floxed *Dlk1* mice were crossed with a Cre driver allele under the control of the *Myf5* promoter. Myf5 is an early critical muscle regulatory factor[32], so *Dlk1-*flox/*Myf5-*Cre mice have their *Dlk1* gene excised during early embryonic development of muscle progenitor cells.

By measuring the expression levels of *Dlk1* in muscle-specific knock-out mice, it was observed that *Dlk1* levels were only decreased by 35% in whole muscle compared to wild-type siblings. However, when *Dlk1* was measured on isolated myofibers and myoblasts, the levels were greatly decreased in muscle-specific mutants, similar to data from *Dlk1-*null mice. Further investigation showed the Dlk1 protein is still detectable in mutant whole muscles originating from non-myogenic cells, mostly endothelial CD31^+^ cells. This is consistent with a recent study that reports many Dlk1^+^ cells found in adult muscle are mesenchymal or fibroblastic in nature, rather than myogenic [17]. However, our data do not indicate that Dlk1 is normally present in satellite cells, contrary to findings by Andersen and others [17]. Our findings suggest that Dlk1 from neighboring interstitial cells or myofibers [2], [4] interacts with satellite cells to influence their physiological state.

The total number of muscle fibers was significantly reduced in our conditional mutant mice. This may account for decreased total body mass that is observed when comparing *Dlk1* cKO mice to wild-type littermates. This phenotype was not expected since the over-expression of Dlk1 does not change the number of myofibers present, only their size and metabolic functions [25]. One possibility is that by removing Dlk1 early in myogenic progenitor cells, the formation of primary and/or secondary myotubes in the embryo may be compromised.

Because of fiber type changes observed in *Dlk1* over-expression models, myosin heavy chain isoforms were measured by qPCR and immuohistochemistry in our *Dlk1* cKO mutants. The mRNA levels of *myosin heavy chain type IIB* were significantly reduced in both the soleus and EDL muscles of mutant mice, while no other isoforms appeared to change. This is consistent with *Dlk1*over-expression models where the expression of type IIB myosin heavy chain was found to be increased in both sheep and mice [22], [25], [65]. This suggests that Dlk1 may play a role in either contraction speed or energy metabolism in the muscle.

The role of Dlk1 in muscle regeneration was observed by challenging muscles with a cardiotoxin (CTX) injury. We found that muscle regeneration was impaired at several levels in the *Dlk1* cKO mice. Myogenin levels and the number of nascent *de novo* fibers were significantly reduced, while cellular infiltrate and levels of pro-inflammatory cytokines were greatly increased. Increased levels of TNF-α have been shown to decrease nascent myofiber genesis [72], [73] which along with IL-1β, activate the NF-κB signaling pathway [62], [74], [75]. We observed high levels of NF-κB in mutant regenerating muscle of *Dlk1* cKO mice. Several studies have shown that NF-κB tightly controls myogenesis and specifically inhibits myofiber formation both in vitro and in vivo [61], [76]. Our data show that lack of Dlk1 in skeletal muscle increases inflammatory response after injury and impairs regeneration potentially through increased activation of NF-κB and expression of inflammatory cytokines. It is also possible that the enhanced inflammatory reaction to muscle injury is due to the poor regeneration of the Dlk1 cKO muscle. Furthermore, reduced phosphorylation of Akt in regenerating myofibers of *Dlk1^−^*cKO mice suggests that Dlk1 may be enhancing myofiber regeneration though the activation of this kinase. Alternatively, inhibition in Akt phosphorylation could be a result of less myofiber regeneration in Dlk1 cKO mice.

We provide direct evidence that Dlk1 function to regulate myogenic progenitor cells. Mice possessing the conditional Dlk1 mutation showed higher proportions of self-renewing cells at the expense of fewer proliferating cells (Fig. 4). In opposition, myoblast differentiation is accompanied by higher levels of Dlk1 protein (Fig. 6). More importantly, overexpression of Dlk1 inhibited myoblast proliferation and promoted their differentiation (Fig. 5). These results support a role of Dlk1 in the regulation of the proliferation and differentiation of satellite cells and provide a mechanism by which Dlk1 overexpression leads to muscle hypertrophy as seen in callipyge sheep and transgenic mice [3], [18], [19].

It remains unknown what signals regulates Dlk1 expression in regenerating and nascent myofibers, and the Dlk1 downstream effectors that affect myoblast proliferation and differentiation. Our results are consistent with the notion that Dlk1 acts as an inhibitor for Notch signaling [28], [29], [30]. Notch signaling is known to inhibit MyoD expression and myogenic differentiation, but enhance satellite cell proliferation and self-renewal[77], [78]. Based on this notion, our Dlk1 conditional mutants should have elevated Notch signaling, which would suppress MyoD expression[79]. Our results confirmed this prediction. Conversely, over-expression of Dlk1 should suppress Notch signaling and promote myogenic differentiation, but inhibit myoblast proliferation[80], [81]. Our overexpression studies again support this notion. Interestingly, recent studies have also shown that Notch signaling interacts with the canonical NF-κB pathway. Notch-1 is able to promote the transcription of TNF-α [82], [83], which in turn stimulates the phosphorylation of IκBα, leading to the activation of NF-κB signaling [83]. Notch-1 is also known to affect macrophage and cultured B cell signaling. Wild-type macrophages increase Notch-1 expression after an immune challenge, which then promotes the transcription of pro-inflammatory cytokines such as TNF-α [82]. In Notch-1-null mice, the immune response and NF-κB activity were three times lower in B-cells compared to wild-type cells [84].Thus, the increased immune response seen in our *Dlk1* cKO injured mice is likely the result of increased Notch-1 signaling, which again support the notion that Dlk1 acts to suppress Notch signaling.

In summary, our data provide novel insight into the mechanisms of action of Dlk1 in skeletal muscle. First we show that ablation of Dlk1 in the Myf5-derived myogenic cells leads to both developmental and postnatal growth/regeneration defects in myogenesis. As the conditional mutants should have normal levels of circulating Dlk1 which is mainly produced by the fetal liver, our results suggest that muscle specific membrane-bound Dlk1 is important for normal myogenesis. We next show that lack of Dlk1 in injured muscle increases the inflammatory response, increasing NF-κB signaling, and decreasing Akt activity. These shifts greatly decrease the ability of the injured muscle to regenerate appropriately. We further show that at progenitor cell level, Dlk1 expression inhibits myoblast proliferation but promotes myogenic differentiation. Therefore, the observed up-regulation of Dlk1 in wild-type muscles after injury may act to allow proliferating myoblasts to differentiate and fuse with existing fibers for muscle repair. When Dlk1 is not present in neighboring tissues, myoblasts are shifted towards the self-renewal state while increasing the inflammatory response, thus hindering muscle repair. These results add to our current understanding of the cellular and molecular mechanisms by which Dlk1 regulates skeletal muscle hypertrophy.

## Materials and Methods

### Mice and Animal Care

Generation of *Dlk1*-floxed mice will be described elsewhere (Appelbe et al., in preparation). Briefly, loxP cassettes were inserted into the endogenous Dlk1 locus flanking exons four and five, and the mice were maintained in a C57BL/6 background. Myf5-Cre mice [85] were provided by Dr. Philip Soriano (Mount Sinai School of Medicine, New York, NY). Mice maintenance and experimental use were performed according to protocols approved by the Purdue Animal Care and Use Committee (PACUC protocol # 08-006).

### Muscle Injury and Regeneration

Muscle regeneration studies were performed using cardiotoxin (CTX; Sigma-Aldrich, St. Louis, MO, USA) injections into the TA muscle. Mice were anesthetized using a ketamine-xylazine cocktail, then 25 µL of 10 µM CTX was injected into the right TA muscle. Muscles were then harvested either at 5 days post-injection for peak regeneration activity or 2–3 weeks post-injection to assess the completion of regeneration and repair.

### Whole Muscle Sections and Staining

Whole muscles (TA, EDL, and soleus) were dissected and either frozen immediately in OCT compound or fixed. For fixation, muscles were placed in 4% PFA for 1 h, 100 mM glycine for h, then 30% sucrose overnight at 4°C. Fixed samples were then frozen in OCT compound. Frozen muscles were cut into 10 µm thick cross sections by a Leica CM1850 cryostat. Immunohistochemistry staining of muscle sections were performed as previously described[44]. Specific primary antibodies can be found in Supplemental Table S1. Fluorescent images were captured with a Coolsnap HQ CCD camera (Photometrics, USA) driven by IP Lab software (Scanalytics Inc, USA) using a Leica DM6000 microscope with a 20X objective (NA = 0.70). H–E staining images were captured by a Nikon D90 digital camera installed on a Nikon (Diaphot) inverted microscope. Monochrome images were processed and composited into color images using Photoshop (CS3) software.

### Single Fiber Culture and Staining

Single myofibers were isolated from the EDL muscles by collagenase I (Roche Applied Science) digestion and tituration as previously described [86], [87]. Suspended fibers were cultured on 60 mm horse serum-coated plates in DMEM media supplemented with 10% fetal bovine serum, 0.5% chick embryo extract, and 1% penicillin-streptomycin for three days. Fibers were then fixed in 4% paraformaldehyde (PFA) and stained for MyoD, Pax7, and GFP, as previously described[44].

### Primary Myoblast Isolation and Culture

Primary myoblasts were isolated from hind limb skeletal muscle. Muscles were minced and digested in type I collagenase and dispase B mixture (Roche Applied Science). Cells were then filtered from debri, centrifuged, and cultured in growth media (F-10 Ham's medium supplemented with 20% fetal bovine serum, 4 ng/mL basic fibroblast growth factor, and 1% penicillin-streptomycin) on collagen-coated dishes at 37°C, 5% CO_2_. Differentiation media consisted of DMEM supplemented with 5% horse serum, 1% penicillin-streptomycin

Myoblast cultures testing the presence of recombinant Dlk1 protein were maintained on a bed of 1 mg/mL BD Matrigel (Basement Membrane Matrix; BD Biosciences, San Jose, CA, USA). Experimental cultures also contained 500 ng/mL of recombinant Dlk1 protein (Dlk1(mouse): Fc(human), cat#ALX-201-416-C010; Alexis Biochemicals-Enzo Life Sciences International, Inc. Plymouth Meeting, PA, USA) in the Matrigel bed. Cells were maintained in the same culture media conditions as listed above for proliferation and differentiation.

### Transfection of C2C12 Cells and primary myoblasts

C2C12 cells and primary myoblasts were transfected using the Neon™ Transfection System (Invitrogen, Inc) according to the manufacturer's recommended protocol. Dlk1 or GFP-containing plasmids were transfected at a rate of 2 µg of plasmid DNA in 2×10^5^ cells. C2C12 cells were then plated in 6-well plates and grown in DMEM with 10% FBS, 1% penicillin/streptomycin. Primary myoblasts were grown under identical conditions as described above in the *Primary myoblast isolation and culture* section.

### RNA Isolation and Quantitative PCR Analysis

Tissue samples for RNA were excised after euthanization and either stored in RNAlater (Ambion, Woodlands, TX) for later processing or homogenized immediately for RNA purification using the Qiagen RNeasy Fibrous Tissue Mini Kit (Qiagen, Inc., Valencia, CA). The on-column DNase treatment included in the kit was used to remove any trace amounts of genomic DNA. Purified RNA samples were then quantified by fluorometry (Quant-iT Ribogreen RNA Quantitation Kit, Invitrogen-Molecular Probes, Eugene, OR). Equal amounts of RNA were reverse transcribed using random hexamer primers and M-MLV reverse transcriptase (Invitrogen, Inc., Carlsbad, CA).

Quantitative PCR was performed using the Roche Lightcycler 480 system with Roche SYBR Green Master Mix reagents (Roche Applied Science, Indianapolis, IN). Samples were assayed in duplicate with 80 ng of cDNA per 10 uL reaction. *Ribosomal protein large protein 38 (Rplp38)* was used as the housekeeping gene for all gene expression studies. Primer sequences and PCR conditions are listed in Supplemental Table S2. Fold expression values relative to the wild-type samples within each tissue were calculated using the 2^−ΔΔCT^ method. Statistical significance was determined by ANOVA using the MIXED procedure of SAS (SAS Institute Inc., Cary, NC, USA).

### Western Blots and Electrophoretic Mobility Shift Assay (EMSA)

Levels of different proteins in skeletal muscle were determined by performing Western immunoblotting as described [88], [89]. Briefly, tissues were washed with phosphate-buffered saline (PBS) and homogenized in western blot lysis buffer (50 mM Tris-Cl [pH 8.0], 200 mM NaCl, 50 mM NaF, 1 mM dithiotheritol (DTT), 1 mM sodium orthovanadate, 0.3% IGEPAL, and protease inhibitors). Approximately 100 µg of protein were resolved on each lane on 10–12% SDS-PAGE, electrotransferred onto nitrocellulose membrane, and probed with specific antibodies (Supplemental Table S1) and detected by chemiluminescence. The bands were quantified using ImageQuant TL software (GE Healthcare).

NF-κB activation in skeletal muscle was analyzed by EMSA as previously described [88] with some modifications. In brief, TA muscles isolated from mice were immediately frozen in liquid nitrogen and suspended at 1 mg muscle weight per 18 µl of low salt lysis buffer (10 mM HEPES [pH 7.9], 10 mM KCl, 1.5 mM MgCl_2_, 0.1 mM EDTA, 0.1 mM EGTA, 1 mM dithiothreitol, 0.5 mM phenylmethylsulfonyl fluoride, 2.0 µg/ml leupeptin, 2.0 µg/ml aprotinin, 0.5 mg/ml benzamidine) followed by mechanical grinding using mortar and pastle. Cells in the lysis buffer were allowed to swell on ice for 10 min followed immediately by three cycles of freeze-thaw lysis. The tubes containing the lysed muscle cells were then vortexed vigorously for 10 s, and the lysate was centrifuged for 30 s at 14,000 rpm. The supernatant (cytoplasmic extracts) was removed and saved at −70°C for further biochemical analysis. The nuclear pellet was resuspended in 4 µl of ice-cold high-salt nuclear extraction buffer (20 mM HEPES [pH 7.9], 420 mM NaCl, 1 mM EDTA, 1 mM EGTA, 150 mM MgCl_2_, 25% glycerol, 1 mM dithiothreitol, 0.5 mM phenylmethylsulfonyl fluoride, 2.0 µg/ml leupeptin, 2.0 µg/ml aprotinin, 0.5 mg/ml benzamidine) per mg of original muscle weight and was incubated on ice for 30 min with intermittent vortexing. Samples were centrifuged for 5 min at 4°C, and the supernatant (nuclear extract) was either used immediately or stored at −70°C. The protein content was measured by the method of BioRad (Hercules, CA) protein assay reagent. EMSAs were performed by incubating 20 µg of nuclear extract with 16 fmol of the ^32^P-end-labeled NF-κB consensus oligonucleotide 5′-AGT TGA GGG GAC TTT CCC AGG C-3′ (Promega, Madison, WI) for 15 min at 37°C. The incubation mixture included 2–3 µg of poly dI.dC in a binding buffer (25 mM HEPES [pH 7.9], 0.5 mM EDTA, 0.5 mM dithiothreitol, 1% Nonidet P-40, 5% glycerol, 50 mM NaCl). The DNA-protein complex thus formed was separated from free oligonucleotide on 7.5% native polyacrylamide gel, using buffer containing 50 mM Tris, 200 mM glycine (pH 8.5), and 1 mM EDTA. The gel was dried, and the radioactive bands were visualized and quantitated by a PhosphorImager (GE Healthcare), using ImageQuant software.

## Supporting Information

Figure S1 **Localization and expression of Dlk1 in skeletal muscles.** **A**: Lack of Dlk1 expression in quiescent Pax7+ satellite cells in a cross section of a resting muscle. **B**: Co-localization of Dlk1 with interstitial CD31^+^ endothelial lineage cells. **C**: Up-regulation of Dlk1 expression in newly regenerated myofibers (small caliber fibers with central nuclei) but not in Pax7+ satellite cells in a regenerating muscle 5 days after cardiotoxin treatment. **D**: Relative *Dlk1* mRNA expression in whole muscles, single myofibers isolated from EDL muscle and cultured myoblasts (n  =  18, 4, 2 respectively; * indicates significant difference compared to whole muscle). (TIF)

Table S1 List of antibodies used in this study. (DOC)

Table S2 Quantitative PCR primers. (DOC)
