# Supplementary figures and images for: Dlk1 Is Necessary for Proper Skeletal Muscle Development and Regeneration
Source: PLoS One. 2010 Nov 29;5(11):e15055. doi: 10.1371/journal.pone.0015055 (PMC2993959; doi:10.1371/journal.pone.0015055)

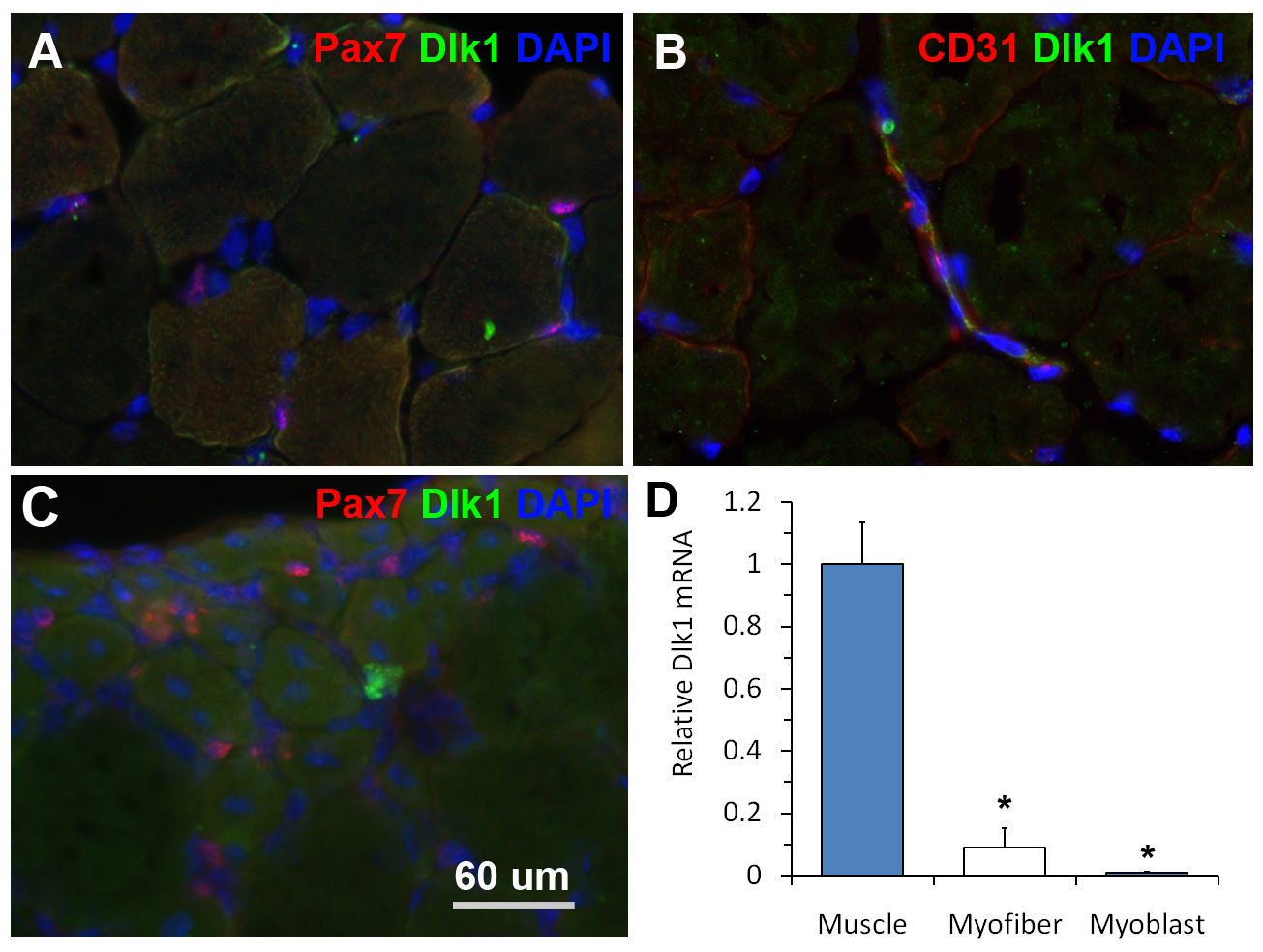

Supplement: Figure S1 — Localization and expression of Dlk1 in skeletal muscles. A: Lack of Dlk1 expression in quiescent Pax7+ satellite cells in a cross section of a resting muscle. B: Co-localization of Dlk1 with interstitial CD31+ endothelial lineage cells. C: Up-regulation of Dlk1 expression in newly regenerated myofibers (small caliber fibers with central nuclei) but not in Pax7+ satellite cells in a regenerating muscle 5 days after cardiotoxin treatment. D: Relative Dlk1 mRNA expression in whole muscles, single myofibers isolated from EDL muscle and cultured myoblasts (n = 18, 4, 2 respectively; * indicates significant difference compared to whole muscle). (TIF) [file pone.0015055.s001.tif]
