# Supplementary material for: Dlk1 Is Necessary for Proper Skeletal Muscle Development and Regeneration
Source: PLoS One. 2010 Nov 29;5(11):e15055. doi: 10.1371/journal.pone.0015055 (PMC2993959; doi:10.1371/journal.pone.0015055)
Supplement: Table S1 — List of antibodies used in this study. (DOC) [file pone.0015055.s002.doc]

Supplemental Table 1: List of antibodies used in this study

| **Antigen** | **Catalog #** | **Company** | **Ig Type** | **Dilution** |
| --- | --- | --- | --- | --- |
| Akt (phosphor) | 9271 | Cell Signaling Technology, Inc. | Rabbit IgG | 1:500 |
| Akt (total) | 9272 | Cell Signaling Technology, Inc. | Rabbit IgG | 1:1000 |
| CD31-PE | 553373 | BD Pharmingen | Rat IgG | 1:100 |
| CD34 | 551387 | BD Biosciences | Rat IgG | 1:100 |
| Dlk1 | Ab21682 | Abcam | Rabbit IgG | 1:100 |
| Dlk1 (H-118) | SC-25437 | Santa Cruz Biotechnology, Inc. | Rabbit IgG | 1:100 |
| GFP | AB13970 | Abcam, Inc. | Chick IgG | 1:2000 |
| IκBα (phosphor) | 9246 | Cell Signaling Technology, Inc. | Mouse IgG1 | 1:500 |
| MyoD, C-20 | SC-304 | Santa Cruz Biotechnology, Inc. | Rabbit IgG | 1:200 |
| Myosin Heavy Chain type IIA | HB-287 | ATCC | Mouse IgG1 | 1:10 culture supernatant |
| Myosin Heavy Chain type I | HB-277 | ATCC | Mouse IgG1 | 1:10 culture supernatant |
| Myosin Heavy Chain type IIB | HB-283 | ATCC | Mouse IgM | 1:10 culture supernatant |
| Pax7 | Pax7 | DSHB, University of Iowa | Mouse IgG1 | 1:10 culture supernatant |
| a-Tubulin | T6074 | Sigma | Mouse IgG1 | 1:3000 |
| VCAM1 (CD106) | 13-1061-85 | eBioscience | Mouse IgG1 biotin-conjugated | 1:100 |
