# Supplementary material for: Dlk1 Is Necessary for Proper Skeletal Muscle Development and Regeneration
Source: PLoS One. 2010 Nov 29;5(11):e15055. doi: 10.1371/journal.pone.0015055 (PMC2993959; doi:10.1371/journal.pone.0015055)
Supplement: Table S2 — Quantitative PCR primers. (DOC) [file pone.0015055.s003.doc]

**Supplemental Table 2: Quantitative PCR primers**

| **Gene name** | **Forward primer (5’-3’)** | **Reverse primer (5’-3’)** |
| --- | --- | --- |
| *β-actin (control)* | CAGGCATTGCTGACAGGATG | TGCTGATCCACATCTGCTGG |
| *CD68* | TTACTCTCCTGCCATCCTTCACGA | CCATTTGTGGTGGGAGAAACTGTG |
| *C-Myc* | CCCCTAGTGCATGAGGA | GCCTCTTCTCCACAGACACC |
| *Dlk1* | CCTGGCTGTGTCAATGGAGT | TGGCAGTCCTTTCCAGAGAA |
| *GFP* | ACGTAAACGGCCACAAGTTC | GCGTGGTAGAAGAAGTTCCT |
| *IL-1β* | CTCCATGAGCTTTGTACAAGG | TGCTGATGTACCAGTTGGGG |
| *MyHC-type I* | AGTCCCAGGTCAACAAGCTG | TTCCACCTAAAGGGCTGTTG |
| *MyHC-type IIA* | AGTCCCAGGTCAACAAGCTG | GCATGACCAAAGGTTTCACA |
| *MyHC-type IIX(d)* | AGTCCCAGGTCAACAAGCTG | CACATTTTGCTCATCTCTTTG |
| *MyHC-type IIB* | AGTCCCAGGTCAACAAGCTG | TTTCTCCTGTCACCTCTCAACA |
| *Myogenin* | CATCCAGTACATTGAGCGCCTA | GAGCAAATGATCTCCTGGGTTG |
| *Numb* | CCGCACTAGAAAGCAAGTCC | ACAAAGTCCCCTTTGCTCCT |
| *Rplp38 (control)* | GAAGGATGCCAAGTCTGTCAA | GAGGGCTGGTTCATTTCAGA |
| *TNF-α* | GCATGATCCGCGACGTGGAA | AGATCCATGCCGTTGGCCAG |
